# Knowledge, attitudes, and practices toward premature ovarian insufficiency: a cross-sectional study among women of childbearing age

**DOI:** 10.3389/fpubh.2025.1685488

**Published:** 2026-01-12

**Authors:** Dong Liu, Shihong Zhang, Qiuyi Wang

**Affiliations:** 1 Department of Obstetrics and Gynecology, West China Second University Hospital, Sichuan University, Chengdu, China; 2 Department of Obstetrics and Gynecology, Hi-Tech Zone Hospital for Women and Children, West China Second University Hospital, Sichuan University, Chengdu, China; 3 Key Laboratory of Birth Defects and Related Diseases of Women and Children (Sichuan University), Ministry of Education, Chengdu, China; 4 Health Management Center, West China Second University Hospital, Sichuan University, Chengdu, China

**Keywords:** cross-sectional study, health behaviors, health literacy, ovarian function decline, perceptions, reproductive health, women's health

## Abstract

**Background:**

Premature ovarian insufficiency (POI) is a significant public health concern, and its early identification in young women is paramount. This study investigated the knowledge, attitude, and practice (KAP) toward POI among women of childbearing age (WOCBA).

**Methods:**

This cross-sectional study was conducted among WOCBA in the primary communities of Chengdu, Sichuan Province, China, from December 2024 to March 2025. A self-designed questionnaire was employed, and participants were selected using a stratified random sampling method.

**Results:**

A total of 459 valid questionnaires were collected, with respondents exhibiting a mean age of 32.44 ± 5.59 years. The majority of participants were married (69.28%), and residing in urban areas (46.19%). The knowledge, attitude, and practice scores were 11.35 ± 10.20 (possible range: 0–38), 37.91 ± 4.29 (possible range: 10–50), and 56.50 ± 15.97 (possible range: 18–90), respectively. Spearman correlation analysis revealed significant positive correlations between knowledge and attitude (*r* = 0.185, *P* < 0.001), between attitude and practice (*r* = 0.474, *P* < 0.001) and between knowledge and practice (*r* = 0.356, *P* < 0.001). Multivariable linear regression analyses showed that both knowledge and attitude were independent predictors of practice scores, and higher educational attainment was also associated with higher practice levels. Living environment contributed significantly to knowledge and attitude scores, while income and family history of early menopause were independently associated with attitude. Structural equation modeling showed that knowledge was associated with attitude (β = 0.210, *P* = 0.015) and with practice (β = 0.301, *P* = 0.010), and attitude was associated with practice (β = 0.431, *P* = 0.005). Knowledge was also indirectly associated with practice through attitude (β = 0.091, *P* = 0.006).

**Conclusion:**

WOCBA demonstrated a constrained understanding of POI, accompanied by moderate attitudes and practices related to it. Targeted educational strategies may help improve awareness of POI among WOCBA, which could be associated with more favorable attitudes and greater engagement in POI-related health-seeking behaviors.

## Introduction

Premature ovarian insufficiency (POI) is a clinical syndrome characterized by the loss or significant decline in ovarian function prior to the age of 40. It is marked by menstrual irregularities, elevated follicle-stimulating hormone (FSH) levels exceeding 25 IU/L, and hypoestrogenism (1, 2). This condition has significant implications for fertility and elevates the risk of long-term health complications, including cardiovascular disease, osteoporosis, fractures, depression, and cognitive decline, ultimately affecting the quality of life (3, 4). Recent research, including a systematic review and meta-analysis published in 2022, estimates the global prevalence of POI to be ~3.5%, reflecting an upward trend over the past two decades (5). The prevalence of POI exhibits regional variation, with reported rates of 3.3% in Asia, 2.3% in Europe, 5.4% in South America, and 11.3% in North America (5). In China, the prevalence is notably higher at 5.3%, compared to 3.1% in developed nations, as reported in the aforementioned 2022 meta-analysis. This discrepancy may arise from genetic, environmental, or healthcare access factors. Other epidemiological studies, however, have reported lower prevalence figures, suggesting that estimates may vary depending on regional sampling methods, diagnostic criteria, and population characteristics. Additional contributing factors such as lifestyle transitions, environmental endocrine disruptors, and variability in healthcare-seeking behaviors may also influence reported prevalence rates. Research indicates that Chinese women with POI face increased mortality and morbidity, as highlighted in a 2014 study (6). These findings underscore the necessity for region-specific investigations, particularly in developing nations such as China.

A critical aspect of understanding POI involves recognizing the influence of individual factors among women. These encompass genetic predispositions, lifestyle choices (including diet, smoking, and stress), and environmental exposures (such as pollutants and chemicals). For example, a 2021 study investigating the genetic causes of idiopathic POI in Chinese Han women identified specific genetic mutations, suggesting a hereditary component (7). Additionally, lifestyle and environmental factors, including exposure to toxins, are implicated, as discussed in a 2021 review on the pathogenesis of POI (8). Understanding these factors is essential for developing targeted interventions that account for regional prevalence variations and inform personalized healthcare strategies.

In China, the large population, evolving reproductive policies (such as the three-child policy), and disparities in healthcare access between urban and rural areas present significant public health challenges (9). The recent implementation of the three-child policy has increased focus on reproductive health; however, public awareness of POI remains limited (10). Cultural factors, including the stigma surrounding infertility, may hinder timely diagnosis and treatment, as indicated by previous study (11). This context highlights the urgency of addressing POI in China, where higher prevalence rates and healthcare disparities exacerbate the condition's impact.

The Knowledge, Attitudes, and Practices (KAP) framework serves as an effective methodology for assessing health literacy, positing that knowledge influences attitudes, which subsequently affect behavior (12). Although KAP studies have primarily concentrated on healthcare providers—revealing that only 53% of gynecologists accurately diagnose POI and fewer than 10% recognize all known causes (13)—there exists a notable gap in research examining KAP among women of reproductive age, particularly in high-prevalence regions like China. Extensive literature searches, including recent studies up to 2025, reveal a lack of comprehensive KAP studies on POI among women of childbearing age (WOCBA) in China, as evidenced by the absence of relevant findings in databases such as PubMed, PMC, and Frontiers (14, 15). This gap underscores the novelty and necessity of the current study, which aims to evaluate KAP regarding POI among WOCBA in China to inform targeted educational and clinical interventions.

## Materials and methods

### Study design and participants

This cross-sectional study was conducted in the primary communities of Chengdu, Sichuan Province, China, from December 2024 to March 2025. The population was categorized into urban and rural strata, with 10 communities randomly selected from each stratum, resulting in a total of 20 communities. The inclusion criteria were as follows: (1) women aged 20–40 years; (2) the ability to read, comprehend, and independently complete electronic questionnaires. The exclusion criteria included: (1) a history of bilateral oophorectomy or hysterectomy; (2) current pregnancy or lactation; and (3) a diagnosis of severe psychiatric disorders (e.g., schizophrenia, bipolar disorder) or neurocognitive impairments (e.g., dementia, intellectual disability) as defined by DSM-5 or ICD-11 criteria, which could impede the ability to complete the questionnaire; (4) age younger than 20 or older than 40 years; and (5) biologically implausible anthropometric data. This study was approved by the Medical Research Ethics Committee of West China Second University Hospital, Sichuan University (Approval No. 470), and written informed consent was secured from all participants to ensure compliance with ethical standards.

### Procedures

A structured, self-designed questionnaire was employed for data collection in this study, guided by pertinent clinical guidelines and existing literature (16–18). The initial iteration of the questionnaire underwent expert review and was revised iteratively based on feedback from four gynecological clinicians, each holding academic positions of associate professor or higher. A preliminary assessment of the questionnaire was conducted with 51 participants, achieving a 100% valid response rate. This assessment yielded a Cronbach's α coefficient of 0.941, with subscale reliability coefficients for the knowledge, attitude, and practice domains recorded at 0.962, 0.715, and 0.931, respectively, indicating satisfactory internal consistency. The final KAP questionnaire consisted of four dimensions: (1) 18 items capturing sociodemographic and physiological background; (2) 19 items assessing knowledge; (3) 10 items measuring attitudes; and (4) 18 items evaluating practices. In the knowledge section, items were scored as follows: “very familiar” (2 points), “heard of it” (1 point), and “unclear” (0 points), resulting in a potential score range of 0–38. The attitude and practice dimensions utilized five-point Likert-type scales, with responses ranked from most to least favorable. For attitude items, entries 1 to 7 and item 9 were scored from 5 to 1 in descending order of agreement, while items 8 and 10 were reverse-coded to reflect negatively phrased content, yielding a total score range of 10–50. All practice items were scored from 5 (most frequent engagement) to 1 (least frequent), resulting in a total possible range of 18–90.

### Questionnaire distribution and quality control

Data were collected utilizing the online Wenjuanxing platform (https://www.wjx.cn/), which was integrated into a WeChat mini-program to facilitate electronic data collection. A unique QR code for the questionnaire was disseminated through WeChat and QQ groups within 20 selected communities (10 urban and 10 rural). Community organizations aided this distribution by sharing the QR code within their online groups, thereby ensuring extensive reach across each community. Each community aimed to gather up to 30 valid questionnaires to maintain regional diversity and balance in the sample. To ensure regional validity, the Wenjuanxing platform restricted IP addresses to users located in Chengdu, permitting each device to submit the questionnaire only once. This strategy prevented duplicate responses and upheld data integrity. All questions were mandatory to minimize missing data and enhance the completeness of responses. Rigorous quality control procedures were implemented, including a manual review of submitted responses for completeness, internal consistency, and logical coherence by trained research staff. Invalid questionnaires were excluded based on specific criteria: completion times under 120 s, patterned responses indicating non-engagement (e.g., identical selections throughout a section), or implausible physiological data, thereby ensuring the reliability of the data.

### Statistical analysis

The sample size was determined prior to data collection to ensure sufficient statistical power for reliable analysis. The questionnaire comprised 47 substantive items spanning the dimensions of knowledge, attitude, and practice. Adhering to the established guideline of 5–10 participants per item (19), this methodology suggested a requisite total sample size ranging from 235 to 470 participants to achieve stable estimates and robust statistical analysis. To account for potential non-responses and incomplete submissions, an additional buffer of 10% was incorporated into the initial target, culminating in a planned recruitment of ~260 to 523 participants.

Statistical analysis was performed using SPSS 26.0 with AMOS (IBM Corp., Armonk, N.Y., USA). The continuous data were tested for normality using the Kolmogorov–Smirnov test. Normally distributed continuous data were expressed as means ± standard deviations (SD) and analyzed using Student's *t*-test (two levels) or ANOVA (more than two levels). Continuous data with skewed distribution were presented as medians (ranges) and analyzed using the Mann–Whitney *U*-test (two levels) or the Kruskal–Wallis test (more than two levels). Categorical variables are expressed as *n* (%). The correlations between KAP dimension scores were assessed using Spearman correlation analysis. Univariate linear regression analyses were first conducted for total knowledge, attitude, and practice scores. Variables with *P* < 0.05 in the univariate analyses were subsequently entered into multivariable linear regression models. Only the results of the multivariable models are presented in the final tables. Furthermore, a structural equation modeling (SEM) analysis was executed to investigate the interrelationships among KAP. Model fit was evaluated utilizing established indices, including the Chi-square to degrees of freedom ratio (CMIN/DF), Root Mean Square Error of Approximation (RMSEA), Incremental Fit Index (IFI), Tucker-Lewis Index (TLI), and Comparative Fit Index (CFI). The hypotheses posited for the SEM analysis were: (1) knowledge directly influences attitude, (2) attitude directly influences practice, and (3) knowledge impacts practice both directly and indirectly (20). All statistical analyses were conducted using a two-sided test, with *P* values < 0.05 deemed statistically significant.

## Results

### Demographic information among women of childbearing age

Of the 580 women who initially accessed the survey, 459 valid responses were retained after exclusions: 37 declined participation, 77 fell outside the designated age range (20–40 years), five had biologically implausible anthropometric data, and two completed the survey in under 120 s. This final cohort had a mean age of 32.44 ± 5.59 years and was predominantly Han Chinese (98.91%), married (69.28%), and urban residents (46.19%). The questionnaire demonstrated high internal reliability (Cronbach's α = 0.949), with a Kaiser-Meyer-Olkin (KMO) measure of sampling adequacy confirming suitability for factor analysis (KMO = 0.949, *P* < 0.001), indicating strong dimensional coherence. Their mean knowledge, attitude, and practice scores were 11.35 ± 10.20 (possible range: 0–38), 37.91 ± 4.29 (possible range: 10–50), and 56.50 ± 15.97 (possible range: 18–90), respectively. Women with higher education (master's degree or above) exhibited superior practice scores (*P* < 0.001). Urban residents demonstrated higher attitude (*P* = 0.014) and practice scores (*P* < 0.001). Participants with monthly incomes ≥5,000 CNY scored significantly higher in attitude (*P* < 0.001) and practice (*P* < 0.001), particularly those earning 5,000–10,000 CNY. Clinical engagement strongly influenced outcomes. Women who underwent AMH testing ( ≤ 1.1 ng/ml) achieved the highest knowledge (*P* = 0.002) and practice scores (*P* < 0.001). Those consulting reproductive specialists reported elevated attitude (*P* < 0.001) and practice scores (*P* < 0.001). Women with familial history of early menopause (< 40 years) showed higher knowledge (*P* < 0.001) but lower attitude scores (*P* = 0.010). However, overweight women exhibited lower practice scores (*P* < 0.001). Married women scored lower in attitude (*P* = 0.035) and practice (*P* = 0.008; Table 1).

**Table 1:** Demographic and clinical characteristics of women of childbearing age and their corresponding KAP scores.

**Variables**	***n* (%)**	**Knowledge, mean ±SD**	***P*-value**	**Attitude, mean ±SD**	***P*-value**	**Practice, mean ±SD**	***P*-value**
	459	11.35 ± 10.20		37.91 ± 4.29		56.50 ± 15.97	
**Tested AMH**			**0.002**		0.076		**< 0.001**
Never tested	352 (76.69)	10.44 ± 9.94		37.81 ± 4.04		55.31 ± 15.87	
Tested, but can't recall the result	68 (14.81)	13.16 ± 9.52		37.71 ± 4.81		57.15 ± 13.4	
Tested, ≤ 1.1ng/ml	15 (3.27)	18.33 ± 13.15		39.87 ± 4.22		67.87 ± 15.22	
Tested, >1.1ng/ml	24 (5.23)	15.25 ± 11.25		38.67 ± 5.95		64.92 ± 19.83	
Age	32.44 ± 5.59						
**BMI (kg/m** ^2^ **)**			0.109		0.144		**< 0.001**
Light	40 (8.71)	9.25 ± 11.24		38.55 ± 4.91		56.55 ± 17.5	
Normal	256 (55.77)	11.07 ± 9.93		38.13 ± 4.17		58.75 ± 15.26	
Obesity	67 (14.60)	12.31 ± 10.92		37.72 ± 4.37		57.58 ± 16.1	
Overweight	96 (20.92)	12.32 ± 9.94		37.2 ± 4.25		49.7 ± 15.43	
**Residence**			0.194		**0.014**		**< 0.001**
Rural	228 (49.67)	10.45 ± 9.58		37.28 ± 4.52		53.11 ± 16.48	
Urban	212 (46.19)	12.42 ± 10.71		38.64 ± 3.91		60.07 ± 14.39	
Suburban	19 (4.14)	10.26 ± 11.08		37.32 ± 4.45		57.32 ± 18.6	
**Education**			0.198		**0.006**		**< 0.001**
Senior high school or below	96 (20.92)	11.91 ± 9.96		36.48 ± 5.03		50.14 ± 16.1	
Associate/bachelor's degree	302 (65.80)	10.63 ± 9.74		38.3 ± 4.06		56.71 ± 15.57	
Master's degree or above	61 (13.29)	14.07 ± 12.27		38.23 ± 3.71		65.44 ± 13.2	
**Monthly income per capita (CNY)**			0.258		**< 0.001**		**< 0.001**
< 2,000	70 (15.25)	11.24 ± 10.17		35.69 ± 4.42		50.31 ± 16.28	
2,000–5,000	161 (35.08)	10.31 ± 9.36		37.93 ± 4.21		52.75 ± 15.36	
5,000–10,000	123 (26.80)	11.29 ± 10.2		38.54 ± 4.33		62.14 ± 15.08	
10,000–20,000	64 (13.94)	14.17 ± 11.21		38.72 ± 3.73		58.7 ± 15.56	
>20,000	41 (8.93)	11.41 ± 11.42		38.46 ± 4		61.41 ± 13.85	
**Type of work**			0.381		**0.012**		**0.013**
Primarily mental work	335 (72.98)	11.09 ± 10.23		38.28 ± 4.05		57.49 ± 15.4	
Primarily physical work	124 (27.02)	12.06 ± 10.14		36.91 ± 4.77		53.81 ± 17.21	
**Work/study environment**			0.091		**0.034**		**< 0.001**
Very comfortable	33 (7.19)	11.15 ± 10.93		38.91 ± 4.87		54.94 ± 19.92	
Comfortable	170 (37.04)	12.47 ± 10.5		38.54 ± 3.82		59.99 ± 15.46	
Average	234 (50.98)	10.25 ± 9.6		37.32 ± 4.36		53.8 ± 15.38	
Poor/very poor	22 (4.79)	14.73 ± 11.98		37.77 ± 5.29		60.5 ± 14.51	
**Living environment**			**0.057**		**< 0.001**		**< 0.001**
Very comfortable	69 (15.03)	14.19 ± 11.73		40.09 ± 4.05		62.93 ± 18.28	
Comfortable	225 (49.02)	11.29 ± 9.82		37.84 ± 3.91		56.84 ± 15.26	
Average and below	165 (35.95)	10.25 ± 9.87		37.08 ± 4.59		53.34 ± 15.11	
**Taken estrogen-progestogen**			0.158		**0.003**		0.181
**medications**							
Yes	103 (22.44)	12.85 ± 10.72		39.11 ± 4.06		58.81 ± 15.12	
No	343 (74.73)	10.73 ± 9.82		37.66 ± 4.23		55.85 ± 16.12	
No, but planning to	13 (2.83)	15.77 ± 14.14		35 ± 5.34		55.23 ± 18.12	
**Marital status**			0.061		**0.035**		**0.008**
Unmarried	124 (27.02)	9.87 ± 10.26		38.81 ± 3.8		59.82 ± 15.21	
Married	318 (69.28)	11.77 ± 10.03		37.64 ± 4.38		55.42 ± 16.11	
Divorced	17 (3.70)	14.29 ± 12.13		36.47 ± 5.11		52.41 ± 15.91	
**Children**			**0.006**		**< 0.001**		**< 0.001**
Yes	298 (64.92)	12.24 ± 10.31		37.37 ± 4.32		54.43 ± 16.02	
No	161 (35.08)	9.71 ± 9.83		38.91 ± 4.06		60.33 ± 15.2	
**Female maternal relatives with**			**< 0.001**		**0.010**		**0.002**
**menopause before 40**							
Yes	27 (5.88)	14.85 ± 11.6		35.63 ± 4.02		56.93 ± 17.76	
No	348 (75.82)	12.02 ± 10.24		38.16 ± 4.23		57.85 ± 15.5	
Don't know	84 (18.30)	7.45 ± 8.55		37.58 ± 4.43		50.74 ± 16.25	
**Consulting ovarian function**			**< 0.001**		**0.011**		**< 0.001**
**information**							
Yes	94 (20.48)	16.19 ± 9.96		38.74 ± 4.71		62.38 ± 14.89	
No	365 (79.52)	10.11 ± 9.9		37.69 ± 4.16		54.98 ± 15.91	
**Diagnosed with premature**			**0.003**		0.922		**0.026**
**ovarian insufficiency**							
Yes	17 (3.70)	18.94 ± 12.24		37.24 ± 6.59		64 ± 18.26	
No	442 (96.3)	11.06 ± 10.02		37.93 ± 4.19		56.21 ± 15.83	
**Consult a reproductive specialist after**			**0.032**		**< 0.001**		**< 0.001**
**premature ovarian insufficiency**							
Yes	123 (26.80)	12.83 ± 10.62		39.08 ± 4.09		64.07 ± 13.92	
No	336 (73.20)	10.81 ± 10.01		37.48 ± 4.29		53.73 ± 15.8	

AMH, anti-Müllerian hormone; BMI, body mass index; CNY, Chinese Yuan; KAP, knowledge, attitude, and practice.

Knowledge score range: 0–38; Attitude score range: 10–50; Practice score range: 18–90. Units: AMH (ng/ml); BMI (kg/m^2^); monthly income (CNY).

Statistical comparisons of continuous variables were performed using Student's t-test or one-way ANOVA for normally distributed data, and Mann–Whitney U-test or Kruskal–Wallis test for non-normally distributed data, as appropriate. No formal statistical power assessment was conducted for subgroup comparisons, as these analyses were descriptive and intended to summarize baseline characteristics rather than to test predefined hypotheses. Imbalance across education categories (96 vs. 302 vs. 61) reflects the underlying distribution of educational attainment in the sampled population and may introduce distributional bias when interpreting subgroup comparisons. Bold values indicate statistical significance.

### Multiple comparisons across subgroups

Pairwise comparisons with Bonferroni-adjusted *P* values were conducted to control for Type I error inflation across multiple subgroup analyses. For AMH testing status, the unadjusted *P* values indicated several significant differences—for example, between “never tested” and “tested ≤ 1.1 ng/ml” for practice (*P* = 0.001)—but these were attenuated after Bonferroni correction (adjusted *P* = 0.007). Similar patterns were observed across BMI categories, where the difference between “normal” and “obesity” remained significant for practice after adjustment (*P* < 0.001). For residence, rural vs. urban differences remained significant for both attitude (adjusted *P* = 0.012) and practice (adjusted *P* < 0.001). Education also showed persistent differences after correction, with “senior high school or below” vs. “associate/bachelor's degree” significant for attitude (adjusted *P* = 0.005) and practice (adjusted *P* = 0.001). Monthly income demonstrated multiple significant adjusted comparisons, particularly between the < 2,000 CNY group and higher-income categories (all adjusted *P* ≤ 0.008). Work/study environment and living environment similarly showed several adjusted significant contrasts, most notably between “very comfortable” and lower-rated environments. Use of estrogen-progestogen medications showed significant adjusted differences between “yes” and both “no” (adjusted *P* = 0.019) and “no, but planning to” (adjusted *P* = 0.020). Marital status differences did not remain significant after adjustment (Table 2).

**Table 2:** Multiple comparisons across subgroups.

**Variables**		**Knowledge**		**Attitude**		**Practice**	
		***P*** **-value**	**Bonferroni** ***P***	***P*** **-value**	**Bonferroni** ***P***	***P*** **-value**	**Bonferroni** ***P***
Tested AMH	Never tested vs. tested, but can't recall result	0.010	0.058			0.232	0.999
	Never tested vs. tested, ≤ 1.1 ng/ml	0.034	0.202			**0.001**	**0.007**
	Never tested vs. tested, >1.1 ng/ml	0.012	0.074			**0.005**	**0.028**
	Tested, but can't recall result vs. tested, ≤ 1.1 ng/ml	0.658	0.999			**0.028**	0.166
	Tested, but can't recall result vs. tested, >1.1 ng/ml	0.268	0.999			**0.039**	0.234
	Tested, ≤ 1.1 ng/ml vs. tested, >1.1 ng/ml	0.521	0.999			0.841	0.999
BMI	Light vs. normal					0.553	0.999
	Light vs. overweight					0.926	0.999
	Light vs. obesity					**0.017**	0.100
	Normal vs. overweight					0.548	0.999
	Normal vs. obesity					**< 0.001**	**< 0.001**
	Overweight vs. obesity					**0.003**	**0.019**
Residence	Rural vs. urban			0.004	**0.012**	**< 0.001**	**< 0.001**
	Rural vs. suburban			0.960	0.999	0.285	0.855
	Urban vs. suburban			0.274	0.822	0.438	0.999
Education	Senior high school or below vs. associate/bachelor			0.002	**0.005**	**< 0.001**	**0.001**
	Senior high school or below vs. master or above			0.037	0.112	**< 0.001**	**< 0.001**
	Associate/bachelor vs. master or above			0.842	0.999	**< 0.001**	**< 0.001**
Monthly income	< 2,000 vs. 2,000–5,000			< 0.001	**0.002**	0.437	0.999
	< 2,000 vs. 5,000–10,000			< 0.001	**< 0.001**	**0.004**	**0.044**
	< 2,000 vs. 10,000–20,000			< 0.001	**0.001**	**0.001**	**0.005**
	< 2,000 vs. >20,000			0.001	**0.008**	**< 0.001**	**< 0.001**
	2,000–5,000 vs. 5,000–10,000			0.462	0.999	**0.010**	0.100
	2,000–5,000 vs. 10,000–20,000			0.387	0.999	**0.001**	**0.011**
	2,000–5,000 vs. >20,000			0.460	0.999	**< 0.001**	**< 0.001**
	5,000–10,000 vs. 10,000–20,000			0.796	0.999	0.340	0.999
	5,000–10,000 vs. >20,000			0.819	0.999	0.094	0.944
	10,000–20,000 vs. >20,000			0.994	0.999	0.710	0.999
Work/study environment	Very comfortable vs. comfortable			0.820	0.999	0.116	0.696
	Very comfortable vs. average			0.088	0.528	0.664	0.999
	Very comfortable vs. poor/very poor			0.601	0.999	0.162	0.970
	Comfortable vs. average			0.007	**0.039**	**< 0.001**	**0.001**
	Comfortable vs. poor/very poor			0.656	0.999	0.704	0.999
	Average vs. poor/very poor			0.438	0.999	**0.037**	0.220
Living environment	Very comfortable vs. comfortable			< 0.001	**< 0.001**	**0.009**	**0.028**
	Very comfortable vs. average and below			< 0.001	**< 0.001**	**< 0.001**	**< 0.001**
	Comfortable vs. average and below			0.256	0.767	**0.042**	0.125
Taken estrogen-progestogen medications	Yes vs. no			0.006	**0.019**		
	Yes vs. no, but planning to			0.007	**0.020**		
	No vs. no, but planning to			0.081	0.243		
Marital status	Unmarried vs. married			0.021	0.063		
	Unmarried vs. divorced			0.070	0.211		
	Married vs. divorced			0.369	0.999		

AMH, anti-Müllerian hormone; BMI, body mass index.

Knowledge score range: 0–38; attitude score range: 10–50; practice score range: 18–90. Units: AMH (ng/ml); BMI (kg/m^2^); monthly income (CNY). Bold values indicate statistical significance.

### Distribution of knowledge, attitude, and practice and correlation analysis

The distribution of knowledge dimensions showed that while 48.15% had heard of POI as ovarian decline before age 40 (**K1**), only 8.06% were very familiar with its definition, and 43.79% remained unsure. Critical deficits were observed in understanding key diagnostic and etiological factors. For instance, merely 8.71% recognized elevated follicle-stimulating hormone (FSH) as a diagnostic marker (**K2b**), with 56.21% uncertain. Similarly, awareness of POI causes was alarmingly low: ≤ 7% correctly identified chromosomal/genetic defects (**K4a**), autoimmune factors (**K4b**), infections (**K4c**), or environmental endocrine disruptors (**K4e**) as contributors, with uncertainty exceeding 57% for all. Therapeutic knowledge was particularly poor, only 8.71% understood the role of hormone replacement therapy (HRT) in mitigating long-term risks (**K5b**), and 52.51% were unsure. Fertility preservation techniques (e.g., oocyte cryopreservation, emerging therapies) were recognized by just 7.41% (**K6c**), with 54.47% expressing uncertainty. Notably, 56.86%−63.18% remained unaware of iatrogenic or environmental risk factors (**K4d–e**). Despite 53.81% acknowledging reduced fertility as a consequence (**K3a**), 49.02% were uncertain about early-stage conception risks (**K2d**; Supplementary Table 1).

Responses to the attitude dimension showed that 21.13% strongly agreed and 42.7% agreed that hormone therapy negatively affects the body's normal endocrine function and is harmful (**A10**), 18.95% strongly agreed and 38.78% agreed that they feel anxious about the possibility of developing premature ovarian insufficiency (**A5**), and 15.03% strongly agreed and 27.02% agreed that being diagnosed with premature ovarian insufficiency would lead to discrimination from people around them (**A8**; Supplementary Table 2). Responses to the practice dimension showed that only 9.59% always seek out information related to premature ovarian insufficiency (**P1**), only 13.07% always raise awareness among family and friends about the harms of premature ovarian insufficiency (**P6**), only 16.12% always choose to undergo ovarian function testing when they have fertility needs (**P3**; Supplementary Table 3). Spearman correlation analysis revealed that significant positive correlations were found between knowledge and practice (*r* = 0.185, *P* < 0.001), knowledge and practice (*r* = 0.356, *P* < 0.001), and attitude and practice (*r* = 0.474, *P* < 0.001), respectively (Table 3).

**Table 3:** Spearman correlation analysis.

**Dimensions**	**Knowledge**	**Attitude**	**Practice**
Knowledge	1^*^	0.185 (*P*<**0.001**)	0.356 (*P*<**0.001**)
Attitude	0.185 (*P*<**0.001**)	1^*^	0.474 (*P*<**0.001**)
Practice	0.356 (*P*<**0.001**)	0.474 (*P*<**0.001**)	1^*^

^*^Self-correlation.

Correlation analysis was conducted on all 459 participants; knowledge score range: 0–38; attitude score range: 10–50; practice score range: 18–90. Bold values indicate statistical significance.

### Multivariable linear regression analyses

Multivariable linear regression analyses were performed to identify independent factors associated with knowledge, attitude, and practice scores. For knowledge, a “comfortable” living environment (β = −3.219, 95% CI: −5.863 to −0.575, *P* = 0.017), an “average and below” living environment (β = −3.629, 95% CI: −6.386 to −0.872, *P* = 0.010), and consulting ovarian function information (β = −4.418, 95% CI: −6.955 to −1.881, *P* = 0.001) were independently associated with lower scores. For attitude, income levels of 2,000–5,000 CNY (β = 1.265, 95% CI: 0.069–2.460, *P* = 0.039), 10,000–20,000 CNY (β = 1.593, 95% CI: 0.074–3.113, *P* = 0.040), and >20,000 CNY (β = 1.740, 95% CI: 0.051–3.429, *P* = 0.044), education at the master's level or above (β = −0.545, 95% CI: −2.145 to 1.055, *P* = 0.505), and maternal relatives experiencing menopause before 40 years (β = 2.256, 95% CI: 0.609–3.903, *P* = 0.008) were independently associated with higher attitude scores, while a “comfortable” living environment (β = −1.736, 95% CI: −2.832 to −0.640, *P* = 0.002), an “average and below” living environment (β = −2.024, 95% CI: −3.207 to −0.840, *P* = 0.001), and planning to take estrogen–progestogen medications (β = −3.928, 95% CI: −6.265 to −1.590, *P* = 0.001) were associated with lower scores. For practice, both knowledge (β = 0.447, 95% CI: 0.329–0.566, *P* < 0.001) and attitude (β = 1.322, 95% CI: 1.039–1.605, *P* < 0.001) were significant independent predictors, and education at the master's level or above was strongly associated with higher practice scores (β = 10.270, 95% CI: 5.672–14.867, *P* < 0.001; Table 4).

**Table 4:** Multivariable linear regression analyses for KAP.

	**Knowledge**		**Attitude**		**Practice**	
	β **(95% CI)**	***P*** **-value**	β **(95% CI)**	***P*** **-value**	β **(95% CI)**	***P*** **-value**
Knowledge			0.079 (0.041–0.118)	**< 0.001**	0.447 (0.329–0.566)	**< 0.001**
Attitude					1.322 (1.039–1.605)	**< 0.001**
**Tested AMH**						
Never tested						
Tested, but can't recall the result	1.738 (−0.858 to 4.334)	0.190				
Tested, ≤ 1.1ng/ml	4.803 (−0.497 to 10.104)	0.076				
Tested, >1.1ng/ml	2.452 (−1.957 to 6.861)	0.276				
Age	0.079 (−0.16 to 0.317)	0.519	−0.029 (−0.13 to 0.072)	0.576		
**Residence**						
Rural						
Urban	1.742 (−0.161 to 3.645)	0.074	0.332 (−0.483 to 1.146)	0.425	1.853 (−0.572 to 4.278)	0.135
Suburban	−0.217 (−4.809 to 4.375)	0.926	−0.287 (−2.183 to 1.609)	0.767	2.319 (−3.437 to 8.075)	0.430
**Education**						
Senior high school or below						
Associate/bachelor's degree			0.336 (−0.746 to 1.417)	0.543	3.628 (0.419–6.838)	**0.027**
Master's degree or above			−0.545 (−2.145 to 1.055)	0.505	10.27 (5.672–14.867)	**< 0.001**
**Monthly income per capita (CNY)**						
< 2,000						
2,000–5,000			1.265 (0.069–2.46)	**0.039**		
5,000–10,000			1.204 (−0.13 to 2.537)	0.078		
10,000–20,000			1.593 (0.074–3.113)	**0.040**		
>20,000			1.74 (0.051–3.429)	**0.044**		
**Type of work**						
Primarily mental wok						
Primarily physical work			0.047 (−0.907 to 1.001)	0.923	1.968 (−0.915 to 4.851)	0.182
**Living environment**						
Very comfortable						
Comfortable	−3.219 (−5.863 to −0.575)	**0.017**	−1.736 (−2.832 to −0.64)	**0.002**	−2.224 (−5.604 to 1.156)	0.198
Average and below	−3.629 (−6.386 to −0.872)	**0.010**	−2.024 (−3.207 to −0.84)	**0.001**	−3.612 (−7.221 to −0.002)	**0.049**
**Taken estrogen-progestogen medications**						
Yes						
No			−0.654 (−1.581 to 0.272)	0.167		
No, but planning to			−3.928 (−6.265 to −1.59)	**0.001**		
**Marital status**						
Unmarried						
Married			0.1 (−1.439 to 1.639)	0.899	1.575 (−2.887 to 6.037)	0.489
Divorced			−0.325 (−2.762 to 2.112)	0.794	−0.401 (−7.642 to 6.84)	0.914
**Children**						
Yes						
No	−1.94 (−4.772 to 0.891)	0.180	1.243 (−0.229 to 2.715)	0.099	2.908 (−1.365 to 7.18)	0.183
Yes						
No			2.256 (0.609–3.903)	**0.008**		
Don't know			2.276 (0.458–4.095)	**0.015**		
**Consulting ovarian function information**						
Yes						
No	−4.418 (−6.955 to −1.881)	**0.001**	−0.802 (−1.796 to 0.192)	0.114	−1.648 (−4.729 to 1.433)	0.295
**Diagnosed with premature ovarian insufficiency**						
Yes						
No	−2.28 (−7.429 to 2.87)	0.386			−0.924 (−7.474 to 5.626)	0.782
**Consult a reproductive specialist after premature ovarian insufficiency**						
Yes						
No			−0.972 (−1.842 to −0.103)	**0.029**	−6.197 (−8.9 to −3.494)	**< 0.001**

AMH, anti-Müllerian hormone; BMI, body mass index; CNY, Chinese Yuan. Bold values indicate statistical significance.

### Structural equation model analysis

**Figure 1:**
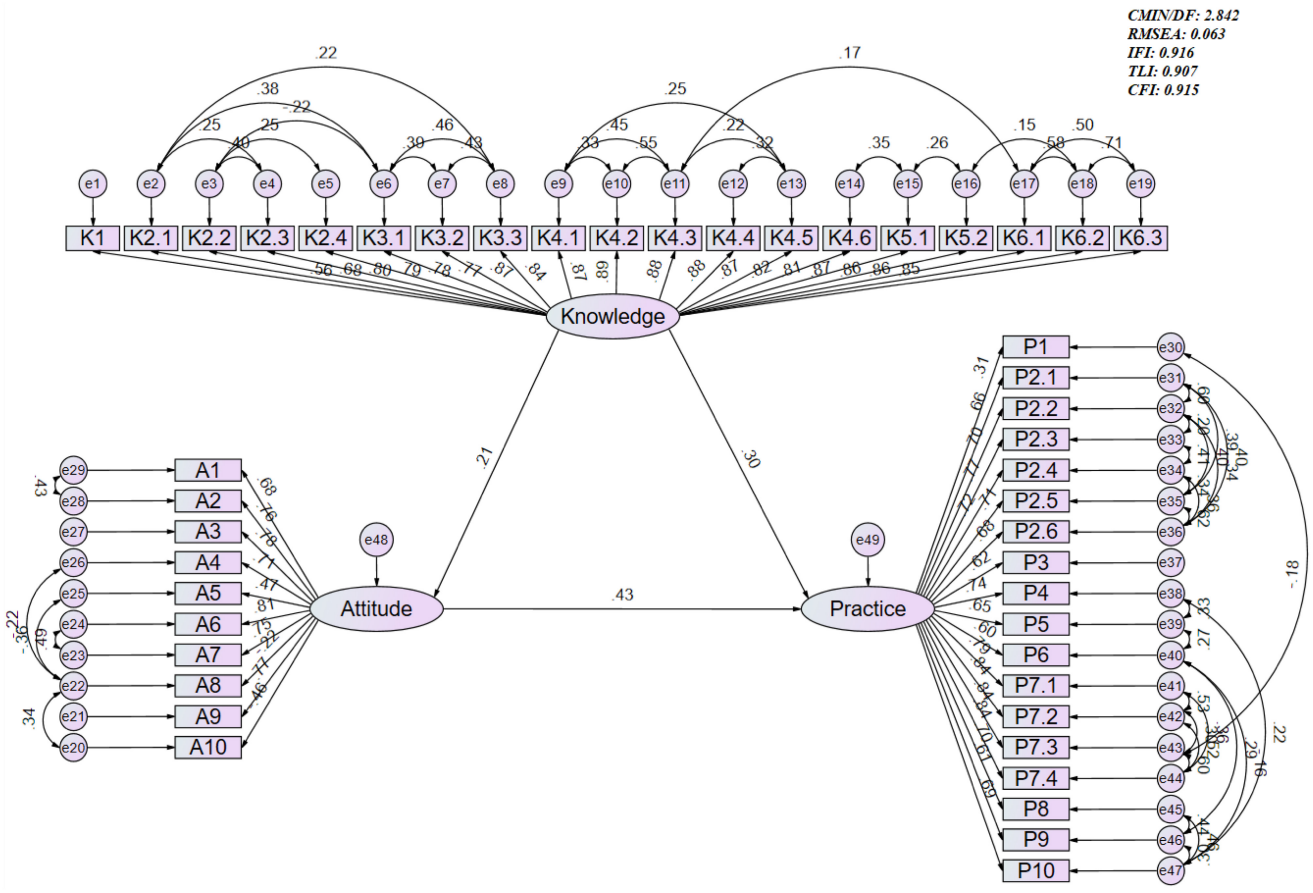
Structural equation model depicting the associations among knowledge, attitude, and practice. The diagram illustrates latent variables (knowledge, attitude, practice) represented by circles, and observed indicators represented by rectangles. Single-headed arrows denote directional associations with standardized coefficients displayed along each path. Double-headed arrows indicate covariances. Error terms for each observed indicator are represented by small circles linked to the corresponding variables.

The final structural equation model demonstrated acceptable to good fit based on multiple fit indices (CMIN/DF = 2.842; RMSEA = 0.063; IFI = 0.916; TLI = 0.907; CFI = 0.915; Supplementary Table 4), and the effect estimates between the various paths have been presented (Supplementary Table 5). The results of SEM direct and indirect effects showed that knowledge had a direct effect on attitude (β = 0.210, 95% CI: 0.068–0.320, *P* = 0.015) and on practice (β = 0.301, 95% CI: 0.188–0.427, *P* = 0.010), while attitude had a direct effect on practice (β = 0.431, 95% CI: 0.356–0.523, *P* = 0.005). Furthermore, knowledge indirectly affected practice through attitude (β = 0.091, 95% CI: 0.042–0.155, *P* = 0.006; Table 5 and Figure 1).

**Table 5:** SEM direct and indirect effects.

**Model paths**	**Standardized total effects**		**Standardized direct effects**		**Standardized indirect effects**	
	β **(95% CI)**	***P*** **-value**	β **(95% CI)**	***P*** **-value**	β **(95% CI)**	***P*** **-value**
Knowledge → attitude	0.210 (0.068–0.320)	**0.015**	0.210 (0.068–0.320)	**0.015**		
Knowledge → practice	0.391 (0.252–0.486)	**0.015**	0.301 (0.188–0.427)	**0.010**		
Attitude → practice	0.431 (0.356–0.523)	**0.005**	0.431 (0.356–0.523)	**0.005**		
Knowledge → practice (indirect effect only)					0.091 (0.042–0.155)	**0.006**

The structural equation model was based on data from all 459 participants. Knowledge score range: 0–38; attitude score range: 10–50; practice score range: 18–90. Bold values indicate statistical significance.

## Discussion

WOCBA demonstrated limited knowledge and moderate attitudes and practices toward POI, despite statistically significant but weak-to-moderate associations among the three domains. Targeted educational interventions are warranted to enhance awareness and improve preventive and management practice related to POI among reproductive-aged women.

The current study explored the interrelationships among KAP regarding POI in women of reproductive age, uncovering several findings that may inform both clinical understanding and future intervention strategies. SEM indicated that knowledge was associated with practice both directly and indirectly through its relationship with attitudes, suggesting that cognitive awareness may shape behavioral engagement in part by altering how individuals perceive the significance and manageability of POI. Among all associations examined, the link between attitude and practice demonstrated the relatively higher standardized effect within the model, indicating an association rather than implying a strong or causal relationship, a pattern observed in prior work on chronic disease (21, 22). These results suggest that educational initiatives that address both cognitive and affective components may better align with the observed patterns of association in health decision-making. However, given the low-to-moderate strength of the observed associations, these findings should be interpreted cautiously and do not indicate robust or directional relationships.

The distribution of responses across the KAP components further illustrates the asymmetry between awareness and action. Despite the generally low knowledge scores—particularly concerning etiological and pathophysiological aspects of POI—many participants expressed moderately positive attitudes and a willingness to engage in preventive or management-related behaviors. Similar phenomena have been noted in studies of polycystic ovary syndrome and early menopause, although methodological and contextual differences limit direct comparability (21, 23). However, this partial compensatory effect appears fragile. As shown in the correlation analysis, knowledge had a weak-to-moderate relationship with practice, and its absence may still constrain informed decision-making, especially in domains such as fertility preservation, hormone therapy, or symptom attribution, where misinformation or ambiguity can have lasting consequences.

Sociodemographic analysis revealed systematic variations in KAP scores. Women who had previously consulted reproductive specialists, undergone ovarian reserve testing, or received estrogen-progestogen therapy generally displayed higher knowledge and practice scores. This likely reflects both increased exposure to clinical information and the reinforcing effect of medical engagement on personal health agency. In contrast, those residing in rural areas, with lower education or income levels, or without prior contact with fertility services exhibited consistently lower scores. These patterns mirror findings in broader reproductive health research, where disparities in health literacy, service access, and perceived relevance of care contribute to uneven uptake of preventive measures (24, 25). Nevertheless, the magnitude of these differences in our study was modest, and caution is warranted in extrapolating these patterns to broader populations.

Attitudinal responses displayed a degree of ambivalence. While many participants agreed that POI warrants clinical attention and that ovarian function testing is appropriate for women over 30, there was less consensus around the role and acceptability of hormone replacement therapy. Responses to items concerning social stigma, emotional burden, and trust in non-pharmacological therapies showed substantial dispersion, which may reflect both limited exposure to consistent health messaging and broader uncertainty about the medicalization of age-related reproductive changes. Previous studies on menopause and reproductive aging have described similar tensions, especially in settings where biomedical and sociocultural narratives diverge (26, 27). The absence of uniformly negative sentiment may reflect variability in exposure to POI-related information rather than indicating readiness for specific educational pathways, but the absence of confident endorsement suggests a persistent information gap.

Behavioral patterns were more heterogeneous and appeared more vulnerable to external constraints. Only a small proportion of participants reported consistently engaging in POI-related practices, such as lifestyle adjustment, information-seeking, or symptom monitoring. Most reported occasional or rare involvement, even in behaviors that require minimal resources, such as discussing symptoms with family or attending health education activities. This behavioral pattern has been observed in other reproductive domains where chronicity and uncertainty undermine sustained engagement (5, 8). The relatively high rate of passive attitudes toward fertility testing, and the limited uptake of hormone therapy despite willingness in principle, may reflect multiple structural or personal factors, although the specific reasons cannot be determined from the present data (9, 28). Such barriers are often multifactorial, shaped by interactions between individual knowledge, social norms, and the responsiveness of the healthcare system.

The knowledge items themselves revealed specific blind spots. While a substantial proportion of respondents had heard of POI, familiarity with its definition, hormonal features, or causes remained extremely limited. Less than one-fifth of participants were familiar with key concepts such as follicle-stimulating hormone elevation or estrogen decline, and awareness of genetic, autoimmune, or environmental causes was even lower. These findings are consistent with regional studies on fertility literacy, which have repeatedly identified endocrine conditions as a major area of unmet educational need (9, 29). In this context, the insufficient knowledge base may not merely reflect personal disinterest, but also the lack of accessible and credible information within clinical encounters and public discourse. However, comparisons with prior studies should be made carefully, as differences in population characteristics, measurement tools, and cultural contexts may limit direct alignment with our findings.

Given these findings, targeted educational strategies appear warranted. Educational programs may benefit from addressing both biomedical information and the perceived relevance of POI among younger women. Materials developed for women in their twenties and thirties may help counter the misconception that POI is a rare or remote concern. Additionally, given the mediating role of attitude, communication strategies should address emotional and motivational barriers, including fears around infertility, stigma, or hormonal treatment. Counseling frameworks that acknowledge uncertainty and offer a range of behavioral options may be more effective than prescriptive messaging alone (30, 31).

Efforts to expand ovarian reserve testing, particularly in primary care or community settings, may serve as gateways to early engagement and personalized counseling. Integrating POI-related content into routine gynecological services and reproductive health programs could normalize discussion and foster early detection. At the same time, clinical training for healthcare providers should emphasize how to identify early signs of POI, navigate sensitive conversations, and recommend follow-up interventions tailored to different levels of knowledge and readiness. Professional education efforts might prioritize rural providers and lower-tier facilities, where such issues are often overlooked (32, 33).

This study has several limitations that should be acknowledged. First, as a cross-sectional design, it captures associations at a single time point and cannot establish causal relationships among knowledge, attitudes, and practices. Secondly, the sample was derived from a singular urban location, which may limit the generalizability of the findings to broader populations or alternative geographic regions. Third, self-reported data may be subject to recall bias or social desirability bias, potentially affecting the accuracy of the responses. Additionally, the number of participants recruited from each community was not reported, which may limit the assessment of representativeness across the sampled communities. Furthermore, the final sample did not maintain an equal distribution between rural and urban strata, which may introduce imbalance in comparative analyses. In addition, the sample was predominantly Han Chinese, resulting in limited ethnic diversity despite the intention to capture variation across communities. Moreover, the study did not assess adherence bias, selective losses, or whether community-level data should be analyzed separately or pooled, which may affect the interpretation of the findings. In addition, although multivariable analyses were conducted, residual confounding cannot be excluded. The instrument used in this study did not undergo external validation beyond the pilot testing phase, which may limit measurement precision. The sampling approach relied on voluntary online participation, introducing the possibility of self-selection bias. Finally, although the SEM demonstrated improved fit in the final model, the cross-sectional nature and measurement constraints limit the robustness and generalizability of the structural pathways.

WOCBA demonstrated limited knowledge and moderately engaged attitudes and practices regarding premature ovarian insufficiency, with significant interrelationships among the three dimensions. These findings highlight the need for targeted educational interventions in clinical settings to enhance early awareness, encourage health-seeking / intentions, and facilitate timely medical consultation for POI.

## Data Availability

The original contributions presented in the study are included in the article/Supplementary material, further inquiries can be directed to the corresponding author.
