# Supplementary material for: Knowledge, attitudes, and practices toward premature ovarian insufficiency: a cross-sectional study among women of childbearing age
Source: Front Public Health. 2026 Jan 12;13:1685488. doi: 10.3389/fpubh.2025.1685488 (PMC12832728; doi:10.3389/fpubh.2025.1685488)
Supplement: Supplementary file 1 [file Table_1.docx]

| Questionnaire ID： |
| --- |
| Dear Participant,  We are researchers from XXX Hospital and sincerely invite you to participate in our research project. This study aims to understand the knowledge, attitudes, and practices of women aged 20–40 years regarding primary ovarian insufficiency (POI), in order to provide a basis for developing scientific intervention strategies. The results may help more people and improve their health outcomes in the future. Your participation in this study is entirely voluntary. The study has been approved by the Ethics Review Committee. If you agree to participate, please read the following instructions.  1. Please complete the questionnaire. There are no right or wrong answers—just respond according to your actual situation. You may ask us any questions during the process, and please submit your responses once completed.  2. This study involves a simple questionnaire survey and will not cause harm to your physical or mental health. However, it does include some private questions, such as your gender and age. We will strictly protect your privacy and keep all information confidential. Please feel free to answer.  3. As a participant, you may request information about the study and its progress at any time. If you decide to withdraw, please inform us, and your data will not be included in the research results.  Finally, we sincerely thank you for taking time out of your busy schedule to support our scientific research!  □I have been informed and agree that the data collected may be used for scientific research.  Informed Consent Signature:  Date of Participation: Year Month Day |

| **Part 1 Basic Information** | |
| --- | --- |
| **1. Have you ever tested your Anti-Müllerian Hormone (AMH) level, and what was the result?** | a. Never tested b. Tested, but can’t recall the result c. Tested, ≤1.1 ng/ml d. Tested, >1.1 ng/ml |
| **2. Your age: _____ years** | |
| **3. Your height: _____ cm** | |
| **4. Your weight: _____ kg** | |
| **5. Where are you from?** | a. Rural area b. Urban area c. Suburban area |
| **6. Your highest education level:** | a. Primary school or below b. Junior high school c. Senior high school / Technical secondary school d. Associate degree / Bachelor’s degree e. Master’s degree or above |
| **7. Your ethnicity:** | a. Han b. Ethnic minority |
| **8. What was your household’s average monthly income per person over the past year (including in-kind and rental income)?** | a. <2000  b. 2000-5000  c. 5000-10000  d. 10000-20000  e. >20000 |
| **9. Your type of work:** | a. Primarily mental work b. Primarily physical work |
| **10.** **How would you describe your current work or study environment?** | a. Very comfortable b. Comfortable c. Average d. Poor e. Very poor |
| **11. How would you describe your current living environment?** | a. Very comfortable b. Comfortable c. Average d. Poor e. Very poor |
| **12. Have you ever taken estrogen-progestogen medications?** | a. Yes b. No c. No, but planning to |
| **13. Your marital status:** | a. Single b. Married c. Divorced d. Widowed |
| **14. Do you have children?** | a. Yes b. No |
| **15.** **Have any of your female maternal relatives (including mother, maternal grandmother, aunts, sisters, etc.) experienced menopause before the age of 40?** | a. Yes b. No c. Don't know |
| **16. Have you ever consulted a doctor regarding ovarian function?** | a. Yes b. No |
| **17. Have you ever been diagnosed with premature ovarian insufficiency?** | a. Yes b. No |
| **18. After being diagnosed with premature ovarian insufficiency, would you consult a reproductive specialist to understand your possible fertility options?** | a. Yes b. No |

| **Part 2 Knowledge of Premature Ovarian Insufficiency (POI)** | | | |
| --- | --- | --- | --- |
| 1. Premature ovarian insufficiency refers to a decline in ovarian function before the age of 40 in women, which may progressively develop into premature ovarian failure. | a. Very familiar | b. Heard of it | c. Unclear |
| 2. Manifestations of premature ovarian insufficiency include: |  |  |  |
| a. Irregular menstruation (e.g., amenorrhea or infrequent menstruation) | a. Very familiar | b. Heard of it | c. Unclear |
| b. Elevated follicle-stimulating hormone (FSH) levels | a. Very familiar | b. Heard of it | c. Unclear |
| c. Fluctuating decline in estrogen levels | a. Very familiar | b. Heard of it | c. Unclear |
| d. Decreased fertility or infertility. Occasional ovulation may still occur in the early stages, with a 5%–10% chance of conception; however, the risk of miscarriage and fetal chromosomal abnormalities increases. | a. Very familiar | b. Heard of it | c. Unclear |
| 3. Adverse effects of premature ovarian insufficiency include: |  |  |  |
| a. Reduced fertility and infertility | a. Very familiar | b. Heard of it | c. Unclear |
| b. Long-term low estrogen state | a. Very familiar | b. Heard of it | c. Unclear |
| c. Perimenopausal symptoms in the short term, such as hot flashes, night sweats, discomfort during intercourse, vaginal dryness, sleep disturbances, anxiety, and depression | a. Very familiar | b. Heard of it | c. Unclear |
| 4. Causes of premature ovarian insufficiency: |  |  |  |
| a. Chromosomal and genetic defects, such as abnormalities in sex chromosomes or autosomal genes, and congenital gonadal dysgenesis | a. Very familiar | b. Heard of it | c. Unclear |
| b. Autoimmune-related ovarian damage | a. Very familiar | b. Heard of it | c. Unclear |
| c. Infections, such as mumps, tuberculosis, malaria, chickenpox, cytomegalovirus, herpes simplex virus, and human immunodeficiency virus | a. Very familiar | b. Heard of it | c. Unclear |
| d. Iatrogenic factors, such as ovarian surgery or chemotherapy and radiotherapy (especially hematopoietic stem cell transplantation during childhood) | a. Very familiar | b. Heard of it | c. Unclear |
| e. Exposure to environmental endocrine disruptors, such as bisphenol A, phthalates, polychlorinated biphenyls, and triclosan, which accelerate follicle depletion and endocrine disorders | a. Very familiar | b. Heard of it | c. Unclear |
| f. Unhealthy lifestyle habits such as smoking and drinking, as well as nutritional factors | a. Very familiar | b. Heard of it | c. Unclear |
| 5. General treatments for premature ovarian insufficiency: |  |  |  |
| a. Lifestyle modification: quitting smoking and limiting alcohol, balanced diet, ensuring sufficient intake of vitamin D and calcium, regular exercise, and maintaining a healthy body mass index | a. Very familiar | b. Heard of it | c. Unclear |
| b. Hormone replacement therapy (HRT): aims to alleviate premature estrogen deficiency, prevent cardiovascular disease and osteoporosis, and help prevent reproductive organ atrophy while improving sexual health, sexual psychology, and quality of sex life | a. Very familiar | b. Heard of it | c. Unclear |
| 6. Reproductive treatments for premature ovarian insufficiency: |  |  |  |
| a. Fertility preservation: technologies such as embryo freezing, oocyte cryopreservation, ovarian tissue cryopreservation, as well as emerging techniques including stem cell therapy, in vitro maturation and activation of oocytes, and artificial ovaries, which show promising potential and application value | a. Very familiar | b. Heard of it | c. Unclear |

| **Part 3 Attitudes Toward Premature Ovarian Insufficiency (POI)** | | | | | |
| --- | --- | --- | --- | --- | --- |
| 1. I believe that premature ovarian insufficiency should be taken seriously by women of childbearing age. | a. strongly agree | b. agree | c. neutral | d. disagree | e. strongly disagree |
| 2. Although there are currently no effective preventive measures for POI, quitting smoking (including avoiding secondhand smoke) and avoiding exposure to reproductive toxic substances can reduce the risk of developing POI. | a. strongly agree | b. agree | c. neutral | d. disagree | e. strongly disagree |
| 3. I believe it is necessary for women over 30 who wish to conceive to undergo regular ovarian function testing. | a. strongly agree | b. agree | c. neutral | d. disagree | e. strongly disagree |
| 3. I believe that even if a woman with POI can conceive naturally, she is more likely to experience miscarriage or fetal chromosomal abnormalities. | a. strongly agree | b. agree | c. neutral | d. disagree | e. strongly disagree |
| 4. I feel anxious about the possibility of developing premature ovarian insufficiency myself. | a. strongly agree | b. agree | c. neutral | d. disagree | e. strongly disagree |
| 5. Although regular check-ups cannot effectively prevent POI, they can help relieve symptoms, improve physical and mental well-being, and enhance quality of life. | a. strongly agree | b. agree | c. neutral | d. disagree | e. strongly disagree |
| 6. Although ovarian function cannot be restored in POI patients, hormone replacement therapy can alleviate symptoms caused by low estrogen and help prevent cardiovascular disease and osteoporosis. | a. strongly agree | b. agree | c. neutral | d. disagree | e. strongly disagree |
| 7. I believe that being diagnosed with premature ovarian insufficiency would lead to discrimination from people around me.（N） | a. strongly agree | b. agree | c. neutral | d. disagree | e. strongly disagree |
| 8. I believe that non-pharmacological therapies (e.g., psychological counseling, exercise therapy) are also important in the treatment of POI. | a. strongly agree | b. agree | c. neutral | d. disagree | e. strongly disagree |
| 9. I believe that hormone therapy negatively affects the body’s normal endocrine function and is harmful.（N） | a. strongly agree | b. agree | c. neutral | d. disagree | e. strongly disagree |

| **Part 4 Practices Regarding Premature Ovarian Insufficiency (POI)** | | | | | |
| --- | --- | --- | --- | --- | --- |
| 1. I actively seek out information related to premature ovarian insufficiency. | a. always | b. often | c. sometimes | d. rarely | e. never |
| 2. Regarding daily management of premature ovarian insufficiency, the measures I tend to take include: |  |  |  |  |  |
| a. Genetic counseling: Learning whether there are POI cases in the family and whether genetic factors are involved; | a. always | b. often | c. sometimes | d. rarely | e. never |
| b. Psychological support: Alleviating psychological burden by communicating with family and friends, participating in support groups, or seeking professional counseling; | a. always | b. often | c. sometimes | d. rarely | e. never |
| c. Lifestyle modifications: Balanced diet, moderate exercise, quitting smoking and limiting alcohol, avoiding harmful substances, and stress relief; | a. always | b. often | c. sometimes | d. rarely | e. never |
| d. Calcium and vitamin D supplementation; | a. always | b. often | c. sometimes | d. rarely | e. never |
| e. Hormone replacement therapy (HRT): To maintain regular menstruation, relieve low estrogen symptoms such as hot flashes, night sweats, and insomnia, and prevent osteoporosis; | a. always | b. often | c. sometimes | d. rarely | e. never |
| f. Other treatments: Such as phytoestrogens, traditional Chinese medicine, coenzyme Q10, DHEA, melatonin, etc.; | a. always | b. often | c. sometimes | d. rarely | e. never |
| 3. If I have fertility needs, I will choose to undergo ovarian function testing (e.g., AMH, FSH, etc.). | a. always | b. often | c. sometimes | d. rarely | e. never |
| 4. If I notice declining ovarian function or have high-risk factors for POI, I will actively adjust my lifestyle to protect ovarian function. | a. always | b. often | c. sometimes | d. rarely | e. never |
| 5. If a doctor recommends hormone replacement therapy to improve POI symptoms, I am willing to try it. | a. always | b. often | c. sometimes | d. rarely | e. never |
| 6. I actively raise awareness among family and friends about the harms of premature ovarian insufficiency. | a. always | b. often | c. sometimes | d. rarely | e. never |
| 7. To prevent premature ovarian insufficiency, the measures I take include: |  |  |  |  |  |
| a. Avoiding unnecessary iatrogenic damage to ovarian function, such as surgery, radiotherapy, or chemotherapy; | a. always | b. often | c. sometimes | d. rarely | e. never |
| b. Actively managing autoimmune diseases and adhering to treatment plans; | a. always | b. often | c. sometimes | d. rarely | e. never |
| c. Maintaining a healthy lifestyle, eating well, exercising moderately, avoiding staying up late and overwork, and managing stress; | a. always | b. often | c. sometimes | d. rarely | e. never |
| d. Avoiding environmental factors: such as pesticides, fertilizers, heavy metals, radioactive substances, etc.; | a. always | b. often | c. sometimes | d. rarely | e. never |
| 8. I actively record and report changes in my symptoms and signs to doctors. | a. always | b. often | c. sometimes | d. rarely | e. never |
| 9. I actively participate in health education and early detection/early treatment awareness activities related to POI. | a. always | b. often | c. sometimes | d. rarely | e. never |
| 10. If I notice symptoms suspected to be related to POI, I will seek medical attention immediately. | a. always | b. often | c. sometimes | d. rarely | e. never |

| **Thank you again for filling out our questionnaire, the information you provided will be valuable to us in the future!**  **Thank you for filling out our questionnaire！**  If you have any comments and suggestions on this survey, we would be honored to hear your voice.  Opinions and Suggestions: （optional）  In order to make this questionnaire study effective and to promote the smooth development of future return visits, we would be grateful if you could leave your contact information!  Your phone number: （optional） |
| --- |
