# Supplementary material for: Knowledge, attitudes, and practices toward premature ovarian insufficiency: a cross-sectional study among women of childbearing age
Source: Front Public Health. 2026 Jan 12;13:1685488. doi: 10.3389/fpubh.2025.1685488 (PMC12832728; doi:10.3389/fpubh.2025.1685488)
Supplement: Supplementary file 2 [file Table_2.docx]

**Supplementary Table 1. Distribution of knowledge dimension responses.**

|  | **N (%)** | | |
| --- | --- | --- | --- |
|  | **Very familiar** | **Heard of it** | **Not sure** |
| **1. Premature ovarian insufficiency refers to a decline in ovarian function before the age of 40 in women, which may progressively develop into premature ovarian failure.** | 37 (8.06) | 221 (48.15) | 201 (43.79) |
| **2. Manifestations of premature ovarian insufficiency include:** |  |  |  |
| **a. Irregular menstruation (e.g., amenorrhea or infrequent menstruation)** | 89 (19.39) | 238 (51.85) | 132 (28.76) |
| **b. Elevated follicle-stimulating hormone (FSH) levels** | 40 (8.71) | 161 (35.08) | 258 (56.21) |
| **c. Fluctuating decline in estrogen levels** | 46 (10.02) | 203 (44.23) | 210 (45.75) |
| **d. Decreased fertility or infertility. Occasional ovulation may still occur in the early stages, with a 5%–10% chance of conception; however, the risk of miscarriage and fetal chromosomal abnormalities increases.** | 49 (10.68) | 185 (40.31) | 225 (49.02) |
| **3. Adverse effects of premature ovarian insufficiency include:** |  |  |  |
| **a. Reduced fertility and infertility** | 57 (12.42) | 247 (53.81) | 155 (33.77) |
| **b. Long-term low estrogen state** | 46 (10.02) | 203 (44.23) | 210 (45.75) |
| **c. Perimenopausal symptoms in the short term, such as hot flashes, night sweats, discomfort during intercourse, vaginal dryness, sleep disturbances, anxiety, and depression** | 46 (10.02) | 210 (45.75) | 203 (44.23) |
| **4. Causes of premature ovarian insufficiency:** |  |  |  |
| **a. Chromosomal and genetic defects, such as abnormalities in sex chromosomes or autosomal genes, and congenital gonadal dysgenesis** | 32 (6.97) | 162 (35.29) | 265 (57.73) |
| **b. Autoimmune-related ovarian damage** | 32 (6.97) | 160 (34.86) | 267 (58.17) |
| **c. Infections, such as mumps, tuberculosis, malaria, chickenpox, cytomegalovirus, herpes simplex virus, and human immunodeficiency virus** | 31 (6.75) | 150 (32.68) | 278 (60.57) |
| **d. Iatrogenic factors, such as ovarian surgery or chemotherapy and radiotherapy (especially hematopoietic stem cell transplantation during childhood)** | 35 (7.63) | 163 (35.51) | 261 (56.86) |
| **e. Exposure to environmental endocrine disruptors, such as bisphenol A, phthalates, polychlorinated biphenyls, and triclosan, which accelerate follicle depletion and endocrine disorders** | 30 (6.54) | 139 (30.28) | 290 (63.18) |
| **f. Unhealthy lifestyle habits such as smoking and drinking, as well as nutritional factors** | 53 (11.55) | 217 (47.28) | 189 (41.18) |
| **5. General treatments for premature ovarian insufficiency:** |  |  |  |
| **a. Lifestyle modification: quitting smoking and limiting alcohol, balanced diet, ensuring sufficient intake of vitamin D and calcium, regular exercise, and maintaining a healthy body mass index** | 53 (11.55) | 224 (48.8) | 182 (39.65) |
| **b. Hormone replacement therapy (HRT): aims to alleviate premature estrogen deficiency, prevent cardiovascular disease and osteoporosis, and help prevent reproductive organ atrophy while improving sexual health, sexual psychology, and quality of sex life** | 40 (8.71) | 178 (38.78) | 241 (52.51) |
| 6. Reproductive treatments for premature ovarian insufficiency: |  |  |  |
| **a. Fertility guidance: including coitus guidance and ovulation induction** | 35 (7.63) | 162 (35.29) | 1. 7.08) |
| **b. Assisted conception: including in vitro fertilization-embryo transfer (IVF-ET) and in vitro fertilization-embryo transfer (IVF-ET) with egg donation.** | 35 (7.63) | 173 (37.69) | 251 (54.68) |
| **c. Fertility preservation: technologies such as embryo freezing, oocyte cryopreservation, ovarian tissue cryopreservation, as well as emerging techniques including stem cell therapy, in vitro maturation and activation of oocytes, and artificial ovaries, which show promising potential and application value** | 34 (7.41) | 175 (38.13) | 250 (54.47) |

**_Knowledge score range: 0–38._**

**Supplementary Table 2. Distribution of attitude dimension responses.**

|  | **N (%)** | | | | |
| --- | --- | --- | --- | --- | --- |
|  | **Strongly agree** | **Agree** | **Neutral** | **Disagree** | **Strongly disagree** |
| **1. I believe that premature ovarian insufficiency should be taken seriously by women of childbearing age.** | 235 (51.2) | 166 (36.17) | 47 (10.24) | 9 (1.96) | 2 (0.44) |
| **2. Although there are currently no effective preventive measures for POI, quitting smoking (including avoiding secondhand smoke) and avoiding exposure to reproductive toxic substances can reduce the risk of developing POI.** | 219 (47.71) | 195 (42.48) | 36 (7.84) | 6 (1.31) | 3 (0.65) |
| **3. I believe it is necessary for women over 30 who wish to conceive to undergo regular ovarian function testing.** | 194 (42.27) | 196 (42.7) | 57 (12.42) | 10 (2.18) | 2 (0.44) |
| **4. I believe that even if a woman with POI can conceive naturally, she is more likely to experience miscarriage or fetal chromosomal abnormalities.** | 117 (25.49) | 212 (46.19) | 116 (25.27) | 13 (2.83) | 1 (0.22) |
| **5. I feel anxious about the possibility of developing premature ovarian insufficiency.** | 87 (18.95) | 178 (38.78) | 120 (26.14) | 60 (13.07) | 14 (3.05) |
| **6. Although regular check-ups cannot effectively prevent POI, they can help relieve symptoms, improve physical and mental well-being, and enhance quality of life.** | 148 (32.24) | 237 (51.63) | 63 (13.73) | 11 (2.4) |  |
| **7. Although ovarian function cannot be restored in POI patients, hormone replacement therapy can alleviate symptoms caused by low estrogen and help prevent cardiovascular disease and osteoporosis.** | 126 (27.45) | 242 (52.72) | 81 (17.65) | 9 (1.96) | 1 (0.22) |
| **8. I believe that being diagnosed with premature ovarian insufficiency would lead to discrimination from people around me.** | 69 (15.03) | 124 (27.02) | 108 (23.53) | 120 (26.14) | 38 (8.28) |
| **9. I believe that non-pharmacological therapies (e.g., psychological counseling, exercise therapy) are also important in the treatment of POI.** | 142 (30.94) | 250 (54.47) | 61 (13.29) | 6 (1.31) |  |
| **10. I believe that hormone therapy negatively affects the body’s normal endocrine function and is harmful.** | 97 (21.13) | 196 (42.7) | 126 (27.45) | 36 (7.84) | 4 (0.87) |

**_Attitude score range: 10–50._**

**Supplementary Table 3. Distribution of practice dimension responses.**

|  | **N (%)** | | | | |
| --- | --- | --- | --- | --- | --- |
|  | **Always** | **Often** | **Sometimes** | **Rarely** | **Never** |
| **1. I actively seek out information related to premature ovarian insufficiency.** | 44 (9.59) | 69 (15.03) | 141 (30.72) | 167 (36.38) | 38 (8.28) |
| **2. Regarding daily management of premature ovarian insufficiency, the measures I tend to take include:** |  |  |  |  |  |
| **a. Genetic counseling: Learning whether there are POI cases in the family and whether genetic factors are involved;** | 35 (7.63) | 60 (13.07) | 153 (33.33) | 121 (26.36) | 90 (19.61) |
| **b. Psychological support: Alleviating psychological burden by communicating with family and friends, participating in support groups, or seeking professional counseling;** | 42 (9.15) | 81 (17.65) | 160 (34.86) | 96 (20.92) | 80 (17.43) |
| **c. Lifestyle modifications: Balanced diet, moderate exercise, quitting smoking and limiting alcohol, avoiding harmful substances, and stress relief;** | 70 (15.25) | 122 (26.58) | 148 (32.24) | 73 (15.9) | 46 (10.02) |
| **d. Calcium and vitamin D supplementation;** | 65 (14.16) | 118 (25.71) | 148 (32.24) | 77 (16.78) | 51 (11.11) |
| **e. Hormone replacement therapy (HRT): To maintain regular menstruation, relieve low estrogen symptoms such as hot flashes, night sweats, and insomnia, and prevent osteoporosis;** | 49 (10.68) | 78 (16.99) | 156 (33.99) | 91 (19.83) | 85 (18.52) |
| **f. Other treatments: Such as phytoestrogens, traditional Chinese medicine, coenzyme Q10, DHEA, melatonin, etc.;** | 48 (10.46) | 74 (16.12) | 139 (30.28) | 102 (22.22) | 96 (20.92) |
| **3. If I have fertility needs, I will choose to undergo ovarian function testing (e.g., AMH, FSH, etc.).** | 74 (16.12) | 104 (22.66) | 133 (28.98) | 93 (20.26) | 55 (11.98) |
| **4. If I notice declining ovarian function or have high-risk factors for POI, I will actively adjust my lifestyle to protect ovarian function.** | 127 (27.67) | 161 (35.08) | 101 (22) | 49 (10.68) | 21 (4.58) |
| **5. If a doctor recommends hormone replacement therapy to improve POI symptoms, I am willing to try it.** | 85 (18.52) | 143 (31.15) | 131 (28.54) | 68 (14.81) | 32 (6.97) |
| **6. I actively raise awareness among family and friends about the harms of premature ovarian insufficiency.** | 60 (13.07) | 97 (21.13) | 124 (27.02) | 124 (27.02) | 54 (11.76) |
| **7. To prevent premature ovarian insufficiency, the measures I take include:** |  |  |  |  |  |
| **a. Avoiding unnecessary iatrogenic damage to ovarian function, such as surgery, radiotherapy, or chemotherapy;** | 99 (21.57) | 86 (18.74) | 117 (25.49) | 88 (19.17) | 69 (15.03) |
| **b. Actively treat autoimmune diseases (such as systemic lupus erythematosus, rheumatoid arthritis, autoimmune thyroid diseases, etc.).** | 92 (20.04) | 101 (22) | 121 (26.36) | 76 (16.56) | 69 (15.03) |
| **c. Maintaining a healthy lifestyle, eating well, exercising moderately, avoiding staying up late and overwork, and managing stress;** | 96 (20.92) | 118 (25.71) | 126 (27.45) | 73 (15.9) | 46 (10.02) |
| **d. Avoiding environmental factors: such as pesticides, fertilizers, heavy metals, radioactive substances, etc.;** | 104 (22.66) | 110 (23.97) | 115 (25.05) | 71 (15.47) | 59 (12.85) |
| **8. I actively record and report changes in my symptoms and signs to doctors.** | 115 (25.05) | 138 (30.07) | 96 (20.92) | 75 (16.34) | 35 (7.63) |
| **9. I actively participate in health education and early detection/early treatment awareness activities related to POI.** | 83 (18.08) | 94 (20.48) | 112 (24.4) | 120 (26.14) | 50 (10.89) |
| **10. If I notice symptoms suspected to be related to POI, I will seek medical attention immediately.** | 168 (36.6) | 111 (24.18) | 87 (18.95) | 71 (15.47) | 22 (4.79) |

_Practice score range: 18–90._

**Supplementary table 4. SEM fit indicators.**

| **Model fit indicators** | **Ref.** | **Measured results** |
| --- | --- | --- |
| **CMIN/DF** | 1-3 excellent，3-5 good | 2.842 |
| **RMSEA** | <0.08 good | 0.063 |
| **IFI** | >0.8 good | 0.916 |
| **TLI** | >0.8 good | 0.907 |
| **CFI** | >0.8 good | 0.915 |

**Supplementary table 5. Estimated total effect coefficient.**

| **Variable** | **Direction** | **Dimensions** | **Estimate** | **Standardized Estimate** | **S.E.** | **C.R.** | **P** |
| --- | --- | --- | --- | --- | --- | --- | --- |
| Attitude | <--- | Knowledge | 0.316 | 0.210 | 0.079 | 3.996 | <0.001 |
| Practice | <--- | Attitude | 0.278 | 0.431 | 0.053 | 5.288 | <0.001 |
| Practice | <--- | Knowledge | 0.291 | 0.301 | 0.064 | 4.579 | <0.001 |
| K1 | <--- | Knowledge | 1.000 | 0.561 |  |  |  |
| K2.1 | <--- | Knowledge | 1.329 | 0.682 | 0.115 | 11.517 | <0.001 |
| K2.2 | <--- | Knowledge | 1.475 | 0.803 | 0.116 | 12.709 | <0.001 |
| K2.3 | <--- | Knowledge | 1.476 | 0.793 | 0.117 | 12.625 | <0.001 |
| K2.4 | <--- | Knowledge | 1.482 | 0.775 | 0.119 | 12.455 | <0.001 |
| K3.1 | <--- | Knowledge | 1.417 | 0.770 | 0.114 | 12.398 | <0.001 |
| K3.2 | <--- | Knowledge | 1.631 | 0.872 | 0.123 | 13.299 | <0.001 |
| K3.3 | <--- | Knowledge | 1.556 | 0.839 | 0.119 | 13.021 | <0.001 |
| K4.1 | <--- | Knowledge | 1.538 | 0.871 | 0.116 | 13.287 | <0.001 |
| K4.2 | <--- | Knowledge | 1.591 | 0.895 | 0.118 | 13.474 | <0.001 |
| K4.3 | <--- | Knowledge | 1.534 | 0.880 | 0.115 | 13.359 | <0.001 |
| K4.4 | <--- | Knowledge | 1.585 | 0.877 | 0.119 | 13.333 | <0.001 |
| K4.5 | <--- | Knowledge | 1.508 | 0.868 | 0.114 | 13.261 | <0.001 |
| K4.6 | <--- | Knowledge | 1.542 | 0.816 | 0.120 | 12.826 | <0.001 |
| K5.1 | <--- | Knowledge | 1.522 | 0.812 | 0.119 | 12.785 | <0.001 |
| K5.2 | <--- | Knowledge | 1.609 | 0.872 | 0.121 | 13.299 | <0.001 |
| K6.1 | <--- | Knowledge | 1.547 | 0.856 | 0.117 | 13.167 | <0.001 |
| K6.2 | <--- | Knowledge | 1.540 | 0.858 | 0.117 | 13.187 | <0.001 |
| K6.3 | <--- | Knowledge | 1.526 | 0.850 | 0.116 | 13.113 | <0.001 |
| A10 | <--- | Attitude | -0.791 | -0.461 | 0.087 | -9.097 | <0.001 |
| A9 | <--- | Attitude | 1.005 | 0.770 | 0.069 | 14.541 | <0.001 |
| A8 | <--- | Attitude | -0.488 | -0.223 | 0.109 | -4.467 | <0.001 |
| A7 | <--- | Attitude | 1.048 | 0.747 | 0.074 | 14.079 | <0.001 |
| A6 | <--- | Attitude | 1.128 | 0.811 | 0.074 | 15.144 | <0.001 |
| A5 | <--- | Attitude | 0.918 | 0.468 | 0.099 | 9.230 | <0.001 |
| A4 | <--- | Attitude | 1.071 | 0.707 | 0.079 | 13.491 | <0.001 |
| A3 | <--- | Attitude | 1.162 | 0.781 | 0.079 | 14.705 | <0.001 |
| A2 | <--- | Attitude | 1.065 | 0.762 | 0.056 | 19.020 | <0.001 |
| A1 | <--- | Attitude | 1.000 | 0.678 |  |  |  |
| P1 | <--- | Practice | 1.000 | 0.311 |  |  |  |
| P2.1 | <--- | Practice | 2.248 | 0.659 | 0.359 | 6.258 | <0.001 |
| P2.2 | <--- | Practice | 2.409 | 0.702 | 0.380 | 6.340 | <0.001 |
| P2.3 | <--- | Practice | 2.636 | 0.766 | 0.409 | 6.441 | <0.001 |
| P2.4 | <--- | Practice | 2.464 | 0.722 | 0.387 | 6.373 | <0.001 |
| P2.5 | <--- | Practice | 2.487 | 0.705 | 0.392 | 6.346 | <0.001 |
| P2.6 | <--- | Practice | 2.439 | 0.677 | 0.387 | 6.295 | <0.001 |
| P3 | <--- | Practice | 2.277 | 0.623 | 0.368 | 6.182 | <0.001 |
| P4 | <--- | Practice | 2.413 | 0.738 | 0.377 | 6.399 | <0.001 |
| P5 | <--- | Practice | 2.176 | 0.646 | 0.349 | 6.230 | <0.001 |
| P6 | <--- | Practice | 2.134 | 0.599 | 0.349 | 6.122 | <0.001 |
| P7.1 | <--- | Practice | 3.147 | 0.791 | 0.486 | 6.473 | <0.001 |
| P7.2 | <--- | Practice | 3.223 | 0.840 | 0.494 | 6.531 | <0.001 |
| P7.3 | <--- | Practice | 3.039 | 0.835 | 0.483 | 6.295 | <0.001 |
| P7.4 | <--- | Practice | 3.165 | 0.837 | 0.485 | 6.528 | <0.001 |
| P8 | <--- | Practice | 2.558 | 0.701 | 0.404 | 6.339 | <0.001 |
| P9 | <--- | Practice | 2.273 | 0.612 | 0.369 | 6.153 | <0.001 |
| P10 | <--- | Practice | 2.498 | 0.688 | 0.396 | 6.311 | <0.001 |
